# Animal Doctors and Animal Patients

**Published:** 1899-09

**Authors:** James Weir

**Affiliations:** Owensboro, Ky.


### Animal Doctors and Animal Patients.

BY JAMES WEIR, JR., M. D., OWENSBORO, KY.

When we come to examine the structural anatomy of the higher animals, including man, we see at once that they are related; when we carry our investigations still further and enter the domains of microscopical anatomy and physiology this relationship is strengthened; when we make an exhaustive examination of the mental manifestations that are to be observed in the life-histories of these creatures when in health, this kinship is still more apparent; but when we study the psychical and physical traits of the higher animals when they are suffering with disease, taking ourselves as standards of comparison, we find them to be so strikingly like the mental and physical traits evinced by ourselves under similar conditions that their relationship ceases to be apparent and becomes real, definite, and very close indeed!

And what has been said of the higher animals can be applied with equal truth to many of the lower animals (crustaceans, insects, reptiles, etc.,) which evince malease when attacked by disease, not only through physical agencies, but through physical manifestations as well. In point of fact, many of the lower animals, when sick, show very plainly by their actions that such is the case. Not only are they able to show disease, but they likewise indicate that they know perfectly well how to combat disease.

The honey bee, when attacked by diarrhœa (a disease to which under certain conditions it is very prone) will immediately begin to suck the astringent pieces of the dogwood, poplar, wild cherry or hickory, and will soon effect a cure of this distressing and often fatal malady. Indeed, such is the intelligence of these little creatures that, in winter, when they become sick with this disease, they will readily drink a decoction of wild cherry bark if it be placed in the hive. When we remember that the bee's taste is even more discriminating than our own, and that it is by no means fond of bitter substances, this instance of their intelligence becomes all the more remarkable.

These insects seem to be aware of the fact that filth is a source of disease; hence, when ill in winter, they select a spot, as far from the combs as possible, at which all of the sick members of the hive deposit their dejecta. As soon as warm weather arrives the accumulated filth is removed and the spot carefully cleansed. In summer all excrementitious matter is deposited without the hive.

Again, crawfish are frequently the hosts of innumerable little parasitic leeches (histriobdellæ) which, strange to say, only become parasites and thus harmful to their hosts, when their number has increased to such an extent that they can no longer live natural lives. As long as they are few in number they are of distinct benefit to their host, the crayfish, for they eat the unimpregnated eggs and dead embryos, thus keeping the other eggs and embryos in a healthy state. But as soon as their number becomes so great that the decomposing eggs and embryos are no longer a sufficient food supply, the mutualists become parasites—they begin to devour the healthy eggs and embryos. The crayfish, which carries her eggs beneath her tail, can tell at once when this state of affairs exists, and will straightway set in motion very effective measures for freeing herself from her harmful visitors.

On one occasion I was observing some gravid crayfish in one of my hatching boxes, when I saw one of them crawl out on the sand shelf, and, by turning partly over, expose one side of her egg cluster to the hot sun. After remaining in this position for a minute or two, she gave the other side a sun bath and then retired to her particular corner of the box. The brooding females all have individual places which they regard as home, and which they guard with jealous watchfulness.

I had never before seen a crayfish act in this manner, therefore was at a loss to account for her actions. Soon another and another, and another of these creatures went through a like performance, until there remained but two in the box that had not sunned their eggs. These were removed to another box where there was no sunlight. On examination I discovered that their eggs were covered with histriobdellæ.

One of these crayfish was then returned to her former home, when, after visiting her corner and finding out that none of her sisters had pre-empted her resting place, she at once went to the sunny shelf and exposed her eggs for some little time to the hot rays of the sun. When she started to return to her corner I picked her up and examined her eggs. The parasites were all dead; they had been killed by the hot sunshine!

The crayfish which had not been allowed to submit her eggs to a sun bath failed to hatch out a single egg, while all of the others produced average broods of young!

The histriobdella is one of the most remarkable creatures in the entire animal kingdom. It is a natural contortionist. Van Benedeu says of a European cousin of the American histriobdella, which has its habitat on the tail of the lobster:

"Let us imagine a clown from the circus, with his limbs as far dislocated as possible, we might even say deprived of bones, displaying tricks of strength and activity on a heap of monster cannon balls, which he struggles to surmount; placing one foot formed like an air bladder on one ball, the other foot on another, alternately balancing and extending his body, folding his limbs on each other, or bending his body upwards like a caterpillar of the geometridæ, and we shall then have but an imperfect idea of all the attitudes which it assumes, and which it varies incessantly." I once saw one of these little animals bend its body downwards (between its legs, as it were) and then suddenly, with a quick flirt, turn a complete somersault!

Fowls, like the crayfish, also have a mutualist (Liothe) which is of distinct benefit to them as long as their number is not too great. They assist very materially in the toilet of their host by eating the dead epithelial layers of skin or scarf-skin and other detritus; they also keep the feathers bright and glossy by devouring all the waste material thrown off in the process of growth. Whenever these mutaulists become too numerous, however, they menace the health of their host, which at once proceeds to get rid of them. The sun baths and dust baths that they administer to themselves are usually very effective medicaments, the liothe being either killed or driven off by them.

Many of the higher animals have discovered and use a materia medica that is not recognized by human physicians, and one which seems to be exceedingly efficacious as far as they are concerned. Thus, dogs will seek out and devour the long, lanceolate blades of ceuch grass (triticum repeus) when they are constipated; horses and mules will eat clay when they have "scours;" cattle with the "scratches" have been seen by me to plaster hoof and joint with mud, and then to stand still until the protecting and healing coating dried out and became firm. I saw a cow not long ago break the thin ice on a pond and treat her itching joints to a mud poultice. Several travelers and hunters of big game declare that they have seen elephants in the act of plugging shot holes with moistened clay! Cats, will go miles when they are feeling "under the weather" for a dose of catnip (nepeta). A gentleman recently informed me that, a short time ago, after a severe snow storm, he was hunting rabbits, when he saw his house cat plowing through the deep snow some distance in advance of him. He thought at first that she was out on the same business as himself, i. e., rabbit hunting, but soon concluded that something of much greater importance had impelled her to abandon the warm kitchen on such a cold and inclement day.

He resolved to follow her, and this he did for three miles, until she finally entered a neighbor's garden, where, after scratching in the snow, she soon uncovered a bunch of catnip. This she at once proceeded to devour! Surely a great and abiding faith in medicine must have dwelt in the bosom of this animal!

An acquaintance of mine, a physician, spent the greater portion of last summer at a farm house. One day, while walking with the farmer, the latter called his attention to a sow that was confined in a pen. The farmer explained that the animal had been kicked in the abdomen by a mule and that he feared that she would die. The sow seemed to be in great pain and was continually trying to get out of the pen. The physician suggested that the door be opened; that the sow probably had peritonitis and would die anyhow. "The door was opened and immediately the sow proceeded toward a disused spring, situated some distance from her pen. This spring issued from beneath the roots of a large tree, and, the water being strongly impregnated with iron, was not used for potable purposes and was fenced about with rails.

"When the animal arrived at this fence she stopped and began to grunt, noting which her owner let down the rails. The hog walked through the gap, at once entered the shallow depression which formed the bowl or bed of the spring, and laid down in the cool water. Here she remained for five days without taking food, though corn in abundance was placed within her reach. At the end of this time she emerged from her self-elected sanitarium completely cured."

It would seem that this animal had grasped some of the leading tenets of the hydrotherapists; at least, in her case, the "water cure" was a complete success.

The saliva of mammals, with the single exception of man, seems to have a distinct curative action. Of course much of the beneficial results following the continual licking of wounds by animals is due to the resulting cleanliness; yet, beyond the mere matter of cleanliness, there is an undoubted curative property in their saliva. Dogs, cats, cattle, rodents, monkeys, et al. lick their wounds when they can get at them, and soon effect cures.

It sometimes happens that animals contract wounds on their bodies which they themselves cannot get at; then, as I have frequently observed, some good samaritan in the shape of a fellow dog, cat or monkey will step in and treat the wounds as though they were personal. My dog Toney is surgeon to all of my other dogs, and, what is more to the point, seems to cure his cases much more effectually and in much shorter time than does his rival in the profession—his master!

Toney is a long-bodied, bench-legged, disreputable looking dog. The hair has been scalded from his body in several places, and he is scarred all over his ungainly carcass, but there is no discounting the fact that he is a remarkably successful doctor. Mingo, another of my dogs, contracted a large ulcer at the base of one of his ears, the result of fly-bites. This sore he could not reach, so it became very much inflamed and very painful. Toney soon discovered the trouble and at once began to treat the ulcer. Several times a day, in fact whenever the sore became dry and painful, Mingo would hunt up Toney, and would patiently stand until the latter thoroughly licked the ulcer. Under such treatment the trouble soon disappeared.

Another of my dogs, Rover, got a severe wound on the back of his head during a fight. This wound also became very much inflamed, the dog's head swelling to almost double its natural size.

Under the careful and intelligent care of Toney, who soon became aware of the trouble (I firmly believe that he was consulted by Rover, just as a man would consult a doctor under like circumstances), the swelling rapidly decreased, the wound became healthy, and, in a week or ten days, was entirely healed.

In a state of nature, when surrounded by an inexhaustible materia medica with which, in my opinion, it is intelligently acquainted, the monkey is very often a successful practitioner in the treatment of the various ills to which it is subject. Even in captivity, when handicapped by its surroundings, this creature often shows that it is able to combat certain diseases intelligently, or to successfully treat an injury.

In 1882 there was on exhibition at the St. Louis fair grounds a magnificent specimen of the dog-faced ape, or chacma. This animal was very large and powerful and at all time treacherous, deceitful, and "possessed of the devil," as his keeper often declared. His malignant disposition caused him to be confined in a strong cage and separated from the other monkeys. There was a strong board partition between his cage and that of a number of smaller monkeys of various genera and species which dwelt together in amity and peacefulness—a "happy family."

The chacma discovered a small crack in the board partition, and, by diligent use of his sharp teeth and powerful fingers, had enlarged it until he could thrust his hands through. After he had severely injured one of the smallest monkeys, which he had caught by darting one of his paws through this opening, the attendant stopped the hole by nailing a piece of board over it on the small monkey's side of the partition.

One of the nails came entirely through the boards and protruded an eighth of an inch into the chacma's cage. One day, while this last mentioned creature was dashing about his den in one of his unaccountable fits of rage, he ran against this nail and scratched his shoulder. He stopped at once and began to examine the hurt with his fingers. He then went to a corner of his cage where there was a box of clean sawdust, and, seizing a handful, pressed it on the bleeding scratch. In a few moments the bleeding ceased, and, when the blood dried, there remained over the wound a coating of sawdust and dried blood which effectually protected it against the attacks of flies, consequently it soon healed.

One day in 1889, while visiting the monkey house at Central Park, New York, I saw a capuchin severely burned by a lighted cigarette which some one had given to it. It wrung the smarting paw in agony of suffering for a moment or two, and then thrust it into a platter of bread and milk. The cool mixture at once gave it relief, and it began to play about the cage. In a few minutes, however, the pain returned. The monkey evidently remembered the anæsthetic qualities of the bread poultice, for it went back to the dish and again thrust in the burned hand. What is most wonderful, however, when it again resumed its play it carried a handful of the cool and soothing poultice of bread and milk in the grasp of the injured member!—*Denver Medical Times.*

